# Dissociation of Neural Substrates of Response Inhibition to Negative Information between Implicit and Explicit Facial Go/Nogo Tasks: Evidence from an Electrophysiological Study

**DOI:** 10.1371/journal.pone.0109839

**Published:** 2014-10-16

**Authors:** Fengqiong Yu, Rong Ye, Shiyue Sun, Luis Carretié, Lei Zhang, Yi Dong, Chunyan Zhu, Yuejia Luo, Kai Wang

**Affiliations:** 1 Laboratory of Cognitive Neuropsychology, Department of Medical Psychology, Anhui Medical University, Hefei, China; 2 School of Humanities and Social Sciences, Beijing Forestry University, Beijing, China; 3 Faculty of Psychology, Universidad Autónoma de Madrid, Madrid, Spain; 4 Department of Neurology, the First Affiliated Hospital of Anhui Medical University, Hefei, China; 5 Anhui Mental Health Center, Hefei, China; 6 Institute of Social and affective Neuroscience, Shenzhen University, Shenzhen, China; University of Rome, Italy

## Abstract

**Background:**

Although ample evidence suggests that emotion and response inhibition are interrelated at the behavioral and neural levels, neural substrates of response inhibition to negative facial information remain unclear. Thus we used event-related potential (ERP) methods to explore the effects of explicit and implicit facial expression processing in response inhibition.

**Methods:**

We used implicit (gender categorization) and explicit emotional Go/Nogo tasks (emotion categorization) in which neutral and sad faces were presented. Electrophysiological markers at the scalp and the voxel level were analyzed during the two tasks.

**Results:**

We detected a task, emotion and trial type interaction effect in the Nogo-P3 stage. Larger Nogo-P3 amplitudes during sad conditions versus neutral conditions were detected with explicit tasks. However, the amplitude differences between the two conditions were not significant for implicit tasks. Source analyses on P3 component revealed that right inferior frontal junction (rIFJ) was involved during this stage. The current source density (CSD) of rIFJ was higher with sad conditions compared to neutral conditions for explicit tasks, rather than for implicit tasks.

**Conclusions:**

The findings indicated that response inhibition was modulated by sad facial information at the action inhibition stage when facial expressions were processed explicitly rather than implicitly. The rIFJ may be a key brain region in emotion regulation.

## Introduction

In social context, appropriately express the negative emotion is important for emotion regulation ability [1]–[3]. However, in some scenarios, it is necessary to inhibit inappropriate negative emotion in social communication. The negative emotion may influence the current goal-directed behavior and further affect social function [4]. An impairment of this ability has increasingly been suggested to be involved in cognitive neural mechanisms of the etiology, maintenance and relapse of a range of psychiatric disorders, including depression [5]–[7], anxiety [8] and post-traumatic stress disorder [9].

Sad facial expressions are fundamental negative emotional stimuli that convey important information in social communications [10]. Emotions induced by sad facial expressions influence an individual's ability to inhibit inappropriate behavior. Many psychiatric individuals have disabilities regulating the relationship between sad facial information and response inhibition [11]. For instance, depressed individuals are often characterized by enhanced facilitation and deficient inhibition for sad emotions, which is a stable cognitive vulnerability risk, possibly associated with the occurrence of depression [12]. Moreover, when new mothers had more sad expressions, their infants expressed less joy and spent more time in joint negative affective states [13].

In several neuroimaging studies, neural substrates of facial information interactions with response inhibition have been investigated. ERP studies on emotional Go/Nogo tasks have reported the presence of N2 and P3 components following the onset of emotional Nogo stimuli. These findings are consistent with previous response inhibition studies employing non-emotional stimuli [14]–[17]. N2 has been suggested to relate to conflict detection and monitoring processes, whereas P3 is responsible for conflict resolution and behavior execution [18], [19]. Using a facial go/nogo task, Zhang group detected larger amplitudes and shorter latency at Nogo-P3 stage in both happy and sad conditions which indicated electrophysiological activity was modulated by facial expressions [16]. Todd and colleagues reported that Nogo-N2 was increased after viewing angry faces than happy faces in 4- to 6-year-old children (N = 48) which suggests that facial expressions interact with response inhibition even at early stages [20]. Spatial features were explored with several fMRI and brain injury studies and data indicated that the right inferior frontal cortex (IFC) is a critical brain region involved in general response inhibition [21]–[25]. Furthermore, many studies indicated the rIFC also plays a crucial role in the neural substrate of response inhibition to negative facial information [26]–[30]. A recent ERP study combining standardized low-resolution brain electromagnetic tomography (sLORETA) with spatio-temporal principal component analysis, revealed the precise contribution of the anterior cingulated cortex (ACC), specifically in the P3 stage of the interplay of emotion and response inhibition [31]. These findings indicate that combining ERP and source localization techniques may be suitable tools for revealing the temporal and spatial characteristics of the brain systems underlying response inhibition.

Although negative emotion and response inhibition interactions play critical roles in human social life and many studies have explored their neural basis; whether the neural substrates of emotional response inhibition dissociated between explicit and implicit processing of facial expressions has been largely unexplored. In fact, implicit and explicit facial processing may serve different functions and may have distinct neural substrates. Explicit processing means that facial expression is within the voluntary attention scope and is directly processed, whereas implicit processing means that facial expression is within involuntary attention scope, and therefore incidentally processed. Thus, the attention resource for stimuli processing is distinct between the two conditions. In social situations, effortful explicit interpretation of the meaning of facial expressions may be required to guide an individual's social responses. However, in familiar situations, facial expression processed implicitly may also affect behavior without full cognitive awareness. It has been confirmed that facial expression processed explicitly and implicitly induced distinct emotional intensity in subject reports. Rating pictures was associated with significantly less intensity of sadness than passively viewing pictures, likely because the rating task reduced the activation of related brain regions responsible for an emotional experience [32]. In a subsequent ERP study, who found that although enhanced processing of negative facial expression occurred at perceptual stages irrespective of intention in facial expressions, larger amplitudes of a late positive complex was detected when facial expression was explicitly processed compared to when it was implicitly processed. Thus, processing of facial expressions depended on the participant's intentional state at late stage [33]. fMRI studies found that directly focusing on emotional valence activated more intense neural reactions in the bilateral amygdala and the superior temporal gyrus, both of which are critical for facial processing [34]. Linden and colleague confirmed that implicit expression processing was preserved in schizophrenia patients, but their explicit emotion classification was impaired, and this finding supported a dissociated mechanism between implicit and explicit processing of facial expression [35].

The aim of the present study was to investigate dissociation of the neural substrates of emotional response inhibition between explicit and implicit processing of facial expressions. In previous emotional response inhibition studies, emotional stimuli were either explicitly [27], [36] or implicitly [16], [28], [31] processed, and the attention was not taken into consideration. It is critical to directly determine whether different manipulations of attention levels for negative stimuli processing would bias the validity of response inhibition to negative stimuli. To this end, we developed a modified implicit and explicit emotional Go/Nogo task to investigate how the negative facial information modulates the response inhibition function implicitly and explicitly. Implicit and explicit tasks have been used to manipulate attention resources for facial expression processing [37]–[39]. In the explicit task, participants were asked to make their Go/Nogo decision based on the recognition of emotional categories, i.e., the emotional information was explicitly processed. In the implicit task, the Go and Nogo trials were defined based on the identification of the gender of the face, i.e., the emotional processing was implicit. Furthermore, the stimuli in the explicit or implicit conditions were identical, which precludes interference due to additional stimuli. A combination of ERP and source localization methods was employed to characterize temporal and spatial characteristics of response inhibition to negative stimuli explicitly and implicitly. Because sad facial expressions are evolutionarily salient emotional stimuli and many psychiatric disorders involve dysfunction in modulating the interaction of inhibition and sad facial emotions [11], [40]–[42], we employed sad and neutral facial stimuli as both Go and Nogo signals using a factorial block design. Based on previous studies that reflected higher emotional intensity in explicit tasks, we hypothesized that significant negative emotional effects would occur in inhibition-related ERP components (N2 and P3) for explicit tasks but not for implicit tasks and within inhibition-related brain areas, such as the rIFC and the ACC.

## Materials and Methods

### Subjects

The study was approved by the Ethics Committees of Anhui Medical University. All participants signed an informed consent form for the experiment.

Thirty right-handed adults (15 female) aged 23.2±1.54 years (mean ± SD) were paid to participate in the experiment. All participants were screened for current and past psychiatric and neurological disorders, were free of histories of drug use and had normal or corrected-to-normal vision. In addition, all subjects scored within the normal range on the State-Trait Anxiety Inventories [43] and the Beck Depression Inventory [44].

### Stimuli

Facial stimuli consisting of 40 sad and 40 neutral faces were selected from the native Chinese Facial Affective Picture System, including 20 female and 20 male faces displaying each emotion type. The faces in the Facial Affective Picture System were assessed with a 9-point scale by 100 college students from two colleges in Beijing. The 9-point scale was used to assess the emotion valence and arousal of each picture in the Facial Affective Picture System. For the valence dimension, participants were asked to assess the valence of the picture. For both valence and arousal dimensions, higher grades refer to more positive valence and stronger arousal respectively, and vice versa. We selected stimuli for the present experiment in such a way that they differed significantly in valence from one another, t = 11.65, *P*<0.001 (M±SD, sad: 3.11±0.63, neutral: 4.49±0.41), but were similar in arousal (*P*>0.5). Stimuli were similar to one another in size, background, spatial frequency, contrast grade, brightness, and other physical properties. Each picture was cropped into the shape of an ellipse that incorporated the facial characteristics using Adobe Photoshop 8.0 software. The screen resolution was 72 pixels per inch, and the viewing angle was 5.7×4.6°. The subjects were seated in a soundproof room with their eyes approximately 100 cm from a 17-in screen. All stimuli were displayed in the center of the screen.

### Experimental procedures

The study used the block design method: one block was implicit and the other was explicit. During the implicit task, the participants were instructed to respond immediately after the pictures depicting one gender (Go trials) and to inhibit this response after the other gender (Nogo trial). In the explicit task, the participants were required to respond or inhibit their behavior according to the valence of the facial expression. The participants were asked to complete both of these two tasks separately in two different blocks. The order of the blocks was counterbalanced across participants. Furthermore, each block was sub-divided into two parts in which the facial stimuli were counterbalanced in terms of whether they indicated Go or Nogo trials.

**Figure 1:**
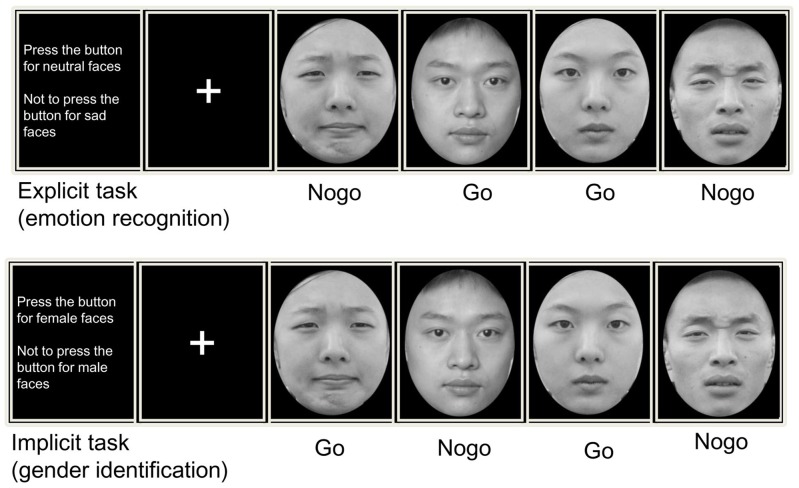
Trial design for (A) an explicit and (B) an implicit emotional Go/Nogo tasks. In explicit task, subjects pressed a response button or inhibit their behavior according to the facial expression (sad/neutral). While in implicit task, subjects made their motor actions based on the facial gender (male/female).

Each block was composed of 480 trials that included 144 Nogo stimuli and 336 Go stimuli (30% vs. 70%). In each block, the Go and Nogo stimuli were presented pseudo-randomly, and the Go trials always preceded the Nogo trials to induce pre-potent motor responses and obvious conflict during response inhibition. At the start of each block, an instruction screen was presented for 2 minutes and prompted the participants to press or refrain from pressing the “J” key with their right hand according to the facial expression or gender. Each trial was initiated by a small grey cross that was displayed for a variable duration (200–400 ms) on the black background. Then, an emotional face was presented at the center of the screen for 1000 ms. Participants were instructed to respond as quickly as possible after the face was presented. Each response was followed by a blank screen, the duration of which varied from 1200 to 1500 ms. The experimental procedure is presented in Figure 1. The individuals in this manuscript have given written informed consent to publish these case details. A training session was included before the formal experiment. All programs were compiled and executed using E-Prime software (Psychology Software Tools, Inc., Pittsburgh, PA).

### Event-related potential recording

Electroencephalography (EEG) was performed from 64 scalp sites using tin electrodes mounted on an elastic cap (Neuro Scan, Sterling, Virginia, USA) according to the international 10/20 system. The participants were grounded with a forehead electrode. All EEG channels were referenced to the left mastoid and were re-referenced off-line to the average of the left and right mastoids. Vertical electro-oculogram (EOG) data were recorded supraorbitally and infraorbitally at the left eye. Horizontal EOG data were recorded as the left versus right orbital rim. EEG and EOG activity were amplified with a 0.01–100 Hz bandpass filter and continuously sampled at 500 Hz/channel. All electrode impedances were maintained below 5 КΩ. Ocular artifacts were removed from the EEG signals using a regression procedure implemented in Neuroscan software [45]. Trials with remaining EOG artifacts (mean EOG voltage exceeding ±100 µV), amplifier clipping artifacts, or peak-to-peak deflections exceeding ±100 µV were excluded from averaging. The EEG activities during correct responses in each condition were aligned and averaged separately. The ERP waveforms were time-locked to the onset of the face stimuli, and the averaging epoch was 1200 ms, including a 200 ms pre-stimulus baseline.

### Data analysis

All behavioral and electrophysiological data analyses were conducted using a SPSS software package (Version 16.0; SPSS Inc, Chicago, USA). The degrees of freedom of the F-ratios were adjusted according to the Greenhouse–Geisser epsilon correction in all analyses. Because this study focused on comparing the effects of the emotion of the stimuli on response inhibition during the implicit and explicit tasks, our analyses mainly concentrated on the interactive effects between task, valence and trial type. Post-hoc contrasts were carried out using the Bonferroni procedure (α<0.05).

### Behavioral analysis

The error rates, defined as Nogo responses in Go trials and button presses in Nogo trials, and the reaction times to Go stimuli were analyzed. Reaction times above 1500 ms or below 150 ms were omitted from the analyses. Repeated-measures ANOVAs were performed on error rates and reaction times using task (implicit and explicit), valence (sad and neutral) and trial type (Go and Nogo) as within-subject factors.

### ERP analysis

With the aim of reliably defining and quantifying N2 and P3 ERP components and increasing the reliability of source analyses, covariance-matrix-based temporal principal component analysis (tPCA) was used in the present study. Albert et al have used this method to analyze N2 and P3 components induced by an emotional Go/Nogo task [31], [46]. This technique has been repeatedly used in ERP researches in that the exclusive use of traditional visual inspection of grand averages and on ‘temporal windows of interest’ for voltage computation may lead to several types of mistake [47]–[49]. The main advantage of tPCA over traditional methods is that it can get ‘clean’ ERP components which are free of the influence of adjacent or subjacent components by extracting and quantifying these components. In fact, the brain potentials recorded at an electrode on the head over a time window represents a complex superposition of different overlapping electrical potentials. Such recordings can interfere with visual inspection. In short, the covariance between all ERP time points tends to be high between those time points involved in the same component and low between those belonging to different components. The tPCA computes the covariance between all ERP time points. Therefore the solution is a set of independent factors composed of highly covariant time points, which ideally correspond to ERP components. Temporal factor score, the tPCA derived parameter in which extracted temporal factors may be quantified is linearly related to amplitude.

As signal overlap may also occur at the spatial domain, the spatial principal component analysis (sPCA) was calculated to reliably define the topography for all temporal factors according to the time window of components obtained by tPCA. The temporal factors (TFs) and spatial factors (SFs) were calculated by tPCA and sPCA steps, respectively, for the components of interest (N2 and P3). In the present study, we selected a number of components based on a screening test [50]. The promax rotation method was used to extract components [47], [48]. Finally, we used repeated measures ANOVAs on these factor scores (linearly related to voltages, as indicated) to analyze the effects of task type, valence and trial type.

### Source-localization analysis

In order to three-dimensionally locate the cortical regions that were sensitive to the experimental effects, standardized low-resolution brain electromagnetic tomography (sLORETA) [51] was applied to relevant temporal factor scores. sLORETA is a three-dimensional discrete linear solution that has been frequently used for EEG source analysis. sLORETA is used to estimate current density distributions restricted to the cortical grey matter and the hippocampus in the digitized MNI atlas with 6239 voxels at a spatial resolution of 5 mm. The sLORETA method has been shown to produce results that coincide with those provided by other brain imaging methods during equivalent paradigms [52]–[55]. Moreover, sLORETA analyses based on temporal factor scores derived from the tPCA method rather than direct voltages have been proven to yield more accurate source-localization results [56]. In addition, the present study employed a relatively large sample size (N = 30) that contributed to reducing the error margin of our EEG-based source analysis.

First, to identify the neural mechanisms underlying response inhibition, voxel-based whole-brain sLORETA images were compared between the Nogo and Go conditions; the sLORETA built-in voxel-wise randomization tests (5000 permutations) based on statistical non-parametric mapping methodology [57] were used. Second, the ROI approach was performed on those regions detected as response inhibition-related in the first step. The current source densities (CSD) of the ROIs were submitted to ANOVA analysis to explore the modulatory effects of task and emotion.

## Results

### Behavioral data

For our behavioral results, both implicit and explicit tasks were relatively easy to perform. The mean accuracy across subjects in each condition was above 95% (95.3% in the negative Nogo condition, 96% in the negative go condition, 97.2% in the neutral Nogo condition, 96.5% in the neutral go condition using an implicit task, 97.5% in the negative Nogo condition, 95.5% in the negative go condition, 98.2% in the neutral nogo condition, and 98.6% in the neutral go condition using an explicit task). The ANOVA analysis of response time in the Go trial type showed that neither the main effects nor the interaction effects were significant (521.3 ms in the negative go condition, 522.3 ms in the neutral go condition using an implicit task; 526.3 ms in the negative go condition, 5201 ms in the neutral go condition using an explicit task).

### ERP data

**Figure 2:**
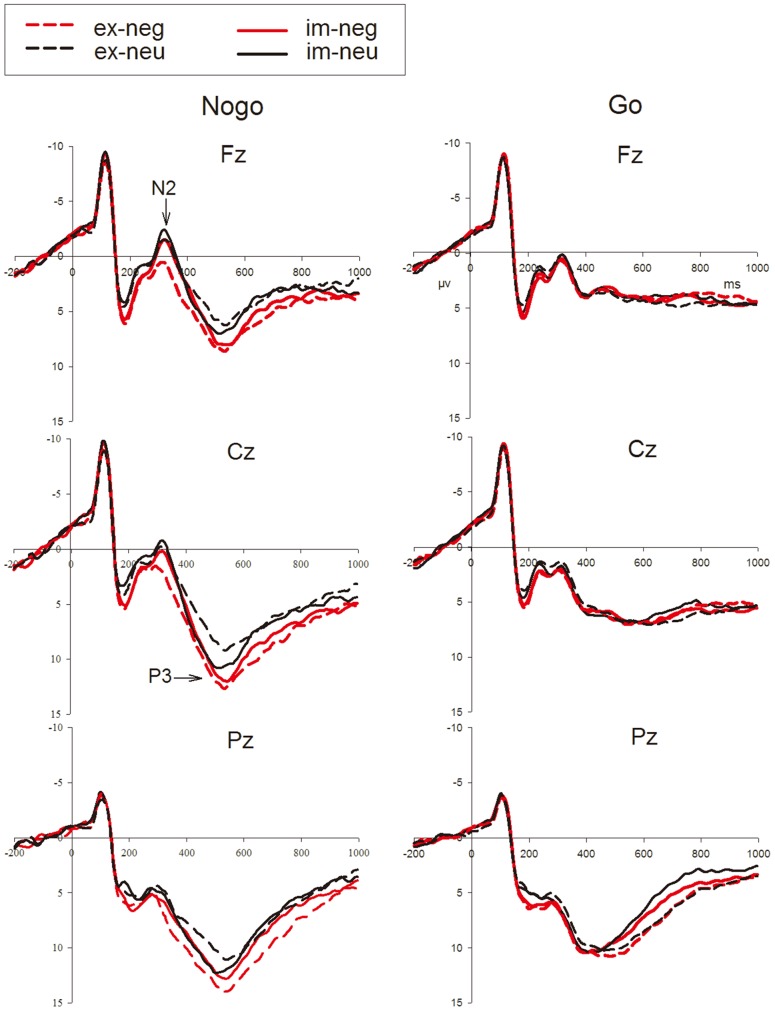
Grand averages evoked by negative (red lines) and neutral (black lines) stimuli in Nogo (left column) and Go trials (right column) under implicit (solid lines) and explicit conditions (dash lines) at Fz, Cz and Pz sites (im: implicit; ex: explicit; neg: negative; neu: neutral).

**Figure 3:**
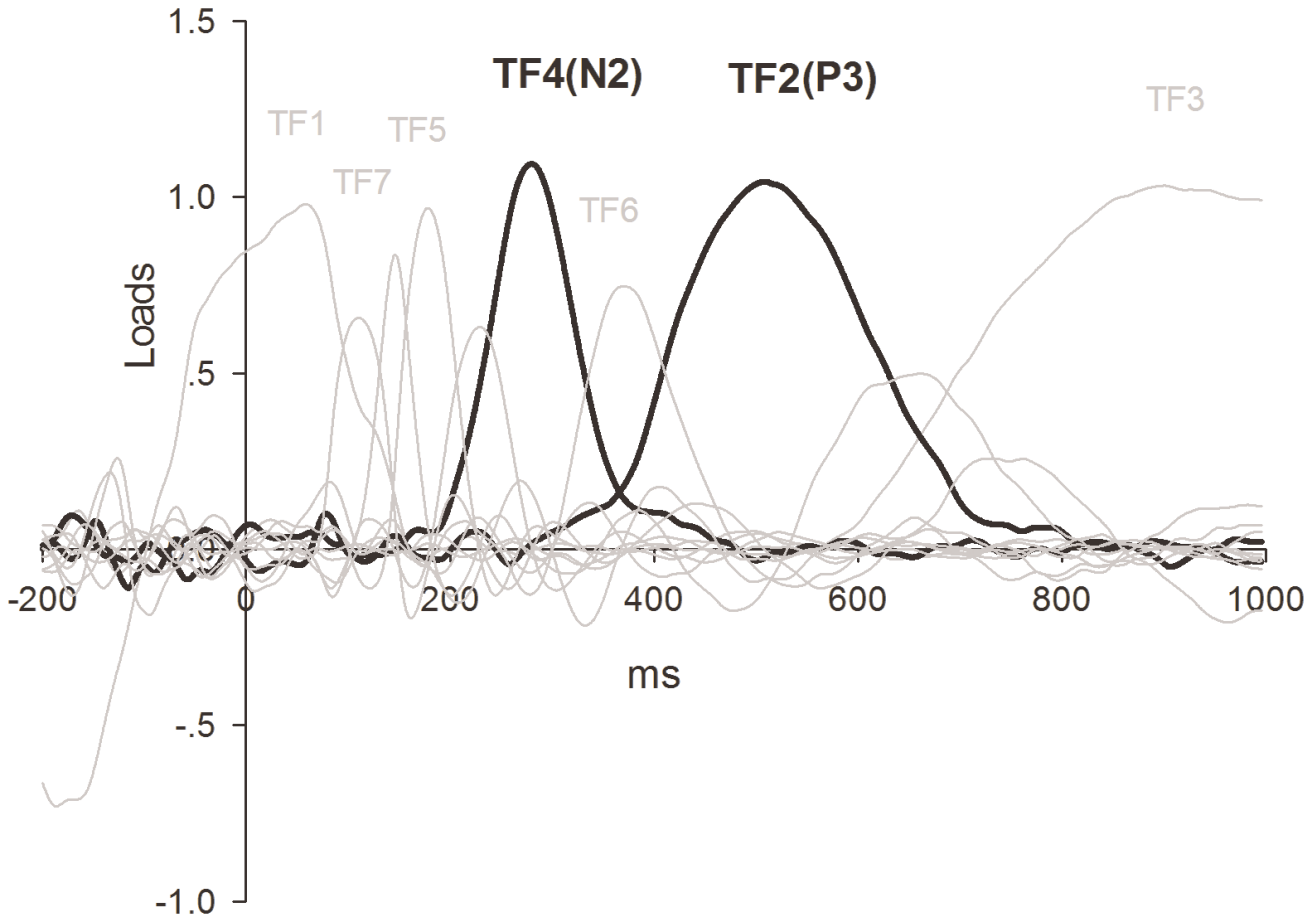
tPCA: factor loadings after promax rotation. TF4 (N2) and TF2 (P3) are drawn in black.

Figure 2 shows the grand averages at the Fz, Cz and Pz sites. As a result of the tPCA computation, seven temporal factors were extracted from the original ERP waveforms (Figure 3). As previous studies revealed that the time window of Nogo-N2 was 200–400 ms and mainly distributed at frontal-central area and Nogo-P3 occur during 400–600 ms and located at central-parietal area [58], [59], we can recognize that TF4 (peak at 284 ms) was associated with the wave labeled N2, and TF2 (peak at 512 ms) was associated with the wave labeled P3 according to factor peak latency and topography characteristics. These labels will be used, henceforth, to make our results easier to understand. The spatial factors extracted by sPCA computations for each temporal factor are shown in Table 1.

**Table 1:** Results of the statistical contrasts concerning Trial type (Go and Nogo) ×Emotional condition (negative, neutral) × Task (implicit, explicit) interaction carried out on N2 and P3 extracted spatio-temporal factors.

Temporal factor	Special factor	trial type	emotion×trial type	task×emotion	task×emotion×trial type
		(df = 1, 29)	(df = 1, 29)	(df = 1, 29)	(df = 1, 29)
TF4 (N2)	Frontocentral	*F* = 10.25**	*F* = 0.69, ns	*F* = 0.24, ns	*F* = 0.65, ns
		*P* = 0.003	*P* = 0.41	*P* = 0.63	*P* = 0.43
	Centroposterior	*F* = 8.22**	*F* = 0.24, ns	*F* = 0.02, ns	*F* = 1.48, ns
		*P* = 0.008	*P* = 0.63	*P* = 0.89	*P* = 0.23
TF2 (P3)	Frontocentral	*F* = 31.61***	*F* = 15.61***	*F* = 3.36, ns	*F* = 2.53, ns
		*P*<0.0001	*P*<0.0001	*P* = 0.08	*P* = 0.12
	Centroposterior	*F* = 17.93***	*F* = 20.48***	*F* = 9.86**	*F* = 4.33*
		*P*<0.0001	*P*<0.0001	*P* = 0.004	*P* = 0.046
	Posterior	*F* = 17.17***	*F* = 3.76	*F* = 16.71***	*F* = 7.25*
		*P*<0.0001	*P* = 0.062	*P*<0.0001	*P* = 0.012

TF: temporal factor; df: degrees of freedom; ns: not significant;

*: *P*<0.05;

**: *P*<0.01;

***: *P*<0.001.

As shown in Table 1, multivariate repeated-measures ANOVAs were computed on N2 and P3 spatial factor scores using task, emotion and trial type as within-subject factors. First, these ANOVA analyses detected significant main effects of trial type in N2 and P3 at the fronto-central and posterior areas. The two components showed higher amplitudes in the Nogo condition than in the Go condition, which further confirmed that they were associated with response inhibition. In addition, temporal factor score of P3 showed significant emotion and trial type interaction effect. Nogo-P3 amplitudes were higher in negative emotion than in neutral conditions. The amplitudes difference induced by go trials between sad and neutral condition was not significant. Moreover, to testify the modulatory effects of task in the Go and Nogo conditions and its further interaction with trial type and emotion, the experimental effects of task ×emotion ×trial type were analyzed. We found a significant interaction of these three factors in the centro-posterior and posterior spatial factors of P3. Post-hoc analyses showed that in the explicit task, Nogo-P3 amplitudes elicited by negative signals were larger than those elicited by neutral signals, while this difference was not found in the implicit task. No interaction effects involving task and/or emotion on trial type were observed in N2 spatial factors.

### Source-localization data

First, source localization analysis consisted of three-dimensionally localizing the cortical regions that were responsible for the experimental manipulations described above. Based on the ERP results of former steps, which detected significant task × emotion × trial type effects in P3 stage, voxel-based whole-brain sLORETA images were compared between the Nogo and Go conditions during the stage using non-parametric randomization tests.

**Figure 4:**
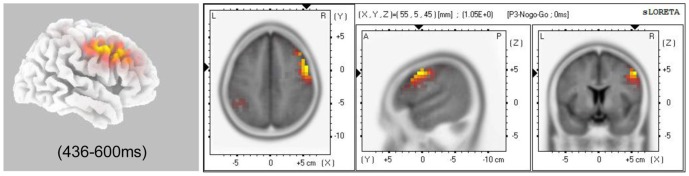
sLORETA solutions to non-parametric randomization tests on P3 temporal factor scores showing voxels in which the Nogo>Go contrast was significant (*P*<0.01).

As illustrated in Figure 4, on P3 stages, Nogo trials compared to Go trials activated the right dorsal lateral prefrontal cortex (rDLPFC, BA 9: max values obtained at x = 50, y = 20, z = 40), the right pre-supplementary motor area (rPre-SMA, BA 6: max values obtained at x = 55, y = 5, z = 45), and the right frontal eye fields (rFEF, BA 8: max values obtained at x = 50, y = 5, z = 45)), which collectively constitute one junction part coinciding with the typical definition of the right inferior frontal junction (rIFJ) [60]–[62]. As explained in Data Analysis, the next step was to define ROIs for each of these data-defined areas, to compute their CSD and to submit them to ANOVAs for testing experimental effects. According to the definition of the rIFJ, one ROI was generated using the coordinates from those voxels surpassing the 0.01 threshold for the t value in the previous sLORETA comparison. We observed a significant main effect of trial type [*F* (1, 29)  = 67.82, *P*<0.0001]. The CSD was higher in the Nogo condition than in the Go condition. More importantly, we detected a significant interaction effect between task and emotion [*F* (1, 29)  = 5.68, *P* = 0.024]. A simple analysis showed that the CSD was larger in the negative condition than in the neutral condition only during the explicit task.

## Discussion

In the present study, we characterized temporal and spatial features of neural substrates of response inhibition to negative faces which were processed explicitly and implicitly. As expected, inhibition-related components, N2 and P3, were successfully induced in the two task sets. The modulatory effect of sad emotion on response inhibition was limited by attention resources during the P3, rather than N2 phase. sLORETA analyses in the P3 stage indicated that—compared to go stimuli—nogo stimuli elicited higher activation in the junctional area of rIFC which consisted of rDLPFC, rPre-SMA and rFEF. ROI analyses confirmed that the three areas were responsible for emotional regulation mechanisms when sad facial emotions interacted with response inhibition explicitly. The implications of these results for understanding emotional response inhibition are discussed below in detail.

Electrophysiological evidence has indicated the diverse functions of N2 and P3 components in response inhibition [19], [63], [64]. Compared to the aspect of conflict response in the N2 stage, previous studies confirmed the action inhibitory process underlying Nogo-P3 [31], [65]. In our study, inhibition related-P3 amplitudes were modulated by sad emotions. Inhibiting sad faces elicited larger P3 amplitudes than neutral faces and these results were consistent with previous studies showing that the capacity of cognitive control could be affected in this predominant fashion when the processing of negative stimuli and emotional significance was sufficient [66]–[68]. Negative emotion, especially a sad facial expression, is biologically and socially significant in human interaction. Human beings often generate withdrawal-related behaviors to negative stimuli which are perceived as threatening information and remind individuals to keep away from the dangerous situation [69]. Thus, when presented with a sad expression, participants devoted more cognitive resources to inhibiting the negative stimuli and elicited larger Nogo-P3 amplitudes than when presented with neutral faces. However, we detect no effect of task, emotion or trial type on behavioral data, most likely because the stimulus parameters were exactly matched between the manipulated variables and the effort of performing the task was similar and simple.

More importantly, we detected discrepancies of Nogo-P3 amplitudes induced by sad and neutral faces between implicit and explicit tasks. P3 amplitudes were larger with sad conditions than with neutral conditions during explicit tasks, but this was not significant during the implicit tasks. Previous studies suggest that individuals with borderline personality disorders have a pronounced withdrawal propensity with explicit tasks; however, implicit behavioral tendencies, measured by the joystick task, were not altered compared to controls [70]. Our results further indicated that response inhibition to facial expression differently between implicit and explicit tasks at P3 stage. Current data were consistent with a study which revealed that adults with ADHD had smaller P3 amplitudes compared to controls in sad conditions and suggested that Nogo-P3 was related to attention allocation [71]. In the explicit task, participants performed their task according to facial expression, and attention was concentrated on negative emotional aspects of faces. In the implicit task, participants were instructed to respond or to inhibit their response according to facial gender, and their attention was diverted from facial expression. Gross has proposed that the emotional effect on cognition is weakened when attention was disengaged from the emotional aspects of stimuli [72], [73]. Thus, our data confirm that the response inhibition to sad faces was modulated by attention resources in the action inhibition stage. This result were also consistent with a previous study in which attention resources modulated facial expression processing and neural activity [32], [33], [74], [75]. McRae group also discovered that the subjective experience and amygdala activity were decreased when attention was diverted from negative emotional pictures to neutral letters [76].

In contrast, Zhang and co-workers reported larger Nogo-P3 amplitudes for emotional conditions than with non-emotional condition [16] in implicit face Go/Nogo tasks. This may be explained by different emotional types used in the study. Happy, fearful, angry faces used in Zhang's work and sad faces have different neural mechanisms and have been reported to have different effects on cognition [27], [77], [78]. Also, shorter face durations of 100–250 ms used in the study may facilitate distinguishing the effect of emotional and neutral faces on response inhibition. Two factors of emotional type and stimuli duration should be further explored to confirm this. We observed no interaction effect of task and emotion with Nogo-N2. Evidence suggests that N2 subcomponents were sensitive to involuntary attention shifting but not voluntary attention regulation [79], [80]. Here, we used block design and participants' responses or response inhibition according to the same instruction presented at the beginning of each task. On this occasion, participants allocated their attention to facial expression or gender voluntarily to perform the task. Thus, the Nogo-N2 was not significantly different between the tasks. In any case, the present study not only showed sad emotion modulated response inhibition but also that this modulatory effect depended on attention resources in the late processing stage of response inhibition. These findings provide some indications for the use of cognitive behavior modification for individuals with anxiety and depression who are often troubled by negative emotion. For example, we can train these individuals to divert their attention from the negative aspects and deliver more attention resources to neutral and positive aspects of daily events.

Using the factor-score-based sLORETA method, source localization analysis on P3 components detected that the rIFJ showed a higher activation level in the Nogo condition compared to the Go condition. The right inferior frontal junction (rIFJ) (BA 6, 8 and 9) was a posterior component of the rIFC localized at the intersection of the inferior frontal sulcus and the precentral sulcus, which comprises parts of the DLPFC, the pre-SMA and the FEF [60], [61]. Consistent results have recently been reported that the rIFJ was the causal source of the top-down modulation underlying selective attention, working memory and response inhibition [81]–[83]. The rIFJ recruitment in P3 phases of response inhibition in the present study may support the implication that this junction of different brain regions is crucial to the response inhibition [60].

Consistent with our ERP findings, the results of ROI analyses revealed that the rIFJ—the general source of inhibition-related components—was more activated in the negative condition compared to the neutral condition during the context of the explicit task, and not the implicit task in the P3 processing period. Previous papers included discussions about the influence of negative emotions on response inhibition separately under explicit and implicit conditions [16], [27], [28], [31], [36]. However, the neural substrate of response inhibition to negative stimuli has been scarcely investigated under the direct manipulation of attention resources. Our results suggested that as the key region of inhibitory processing, rIFJ activity was involved in response inhibition to social sad stimuli. As described above, the rIFJ has recently been reported to be the causal source of the top-down modulation underlying selective attention, working memory, and response inhibition [81]–[83]. With current explicit tasks, attention was concentrated on sad facial expressions which may increase the required sources for inhibitory processing. Thus, more effort from the rIFJ would be required for behavioral monitoring and action inhibition relative to an implicit task. The IFJ has been reported to be involved in emotion reappraisal processing [84]. Combined with these results, the IFJ may be a critical brain area for emotion regulation.

Our data did differ from that of Piguet and colleagues, who found no activation of inhibition-related specific area such as the rIFJ whereas ACC activation decreased when subjects were asked to inhibit response to sad or happy faces according to an expression or gender switching task [85]. This is likely because the tasks in Piguet's work included not only inhibition but also switching tasks and timing, stimuli and task difficulty were different from our study. A question that arises is why the ACC has not shown trial type by emotion effects in the implicit and/or explicit tasks. Indeed, the ACC has been reported to be sensitive to this interaction in several previous studies also employing implicit tasks [28], [31], [46]. The nature of the stimuli may contribute to the explanation of this observed lack of effects. Previous experiments included highly arousing stimuli, such as emotional scenes [31], [46] or emotional words specifically selected as being salient for patients included in the experimental sample [28]. Given that the emotional stimuli employed in our study were not especially arousing (sadness is considered a low arousing negative emotion [86]) and that ACC activity has been shown to be sensitive to the intensity/arousal of emotional stimuli [87], these facts may explain the present results.

Our study has several limitations. First, we only used the sad expressions as experimental stimuli and how the six basic emotions interact in response inhibition in implicit and explicit tasks warrants investigation. Second, although the PCA method proved the precision of spatial orientation, the method was still based on the proposition from a mathematical, but not a physiological, standpoint. In addition, there are many sub-cortical areas involved in response inhibition to facial stimuli such as the amygdala, ventral striatum and fusiform gyrus [27], [71]. But because of the limitation of electrophysiological source analysis, the sub-cortical areas could not be observed. Further researches employing a wide range of experimental tasks and designs, as well as brain imaging methodologies, such as fMRI, that may improve the spatial resolution, are needed to substantiate and extend these findings.

## Conclusions

In summary, using a high temporal resolution of ERPs and recent advances in reconstruction of electrophysiological sources, our data suggest that the neural substrates of response inhibition to sad faces were different between implicit and explicit tasks. The P3 amplitudes were greater in sad compared to neutral conditions in explicit tasks rather than in implicit tasks. Furthermore, the spatial dissociation in brain functions confirmed that the rIFJ was the central node in response inhibition to sad faces. These findings collectively revealed the underlying temporal and spatial characteristics in response inhibition to social negative stimuli explicitly and implicitly, which may have implications for future research concerning the complicated interaction of emotion and cognition and cognitive behavior modification in psychiatry individuals.

## Supporting Information

Information S1 **Spatio-temporal factor score of P3; Spatio-temporal factor score of N2; Current source density of rIFJ calculated by sLORETA analysis on P3 factor.**
(RAR)
